# Is DOPA-Responsive Hypokinesia Responsible for Bimanual Coordination Deficits in Parkinson’s Disease?

**DOI:** 10.3389/fneur.2013.00089

**Published:** 2013-07-19

**Authors:** Quincy J. Almeida, Matt J. N. Brown

**Affiliations:** ^1^Sun Life Financial Movement Disorders Research and Rehabilitation Centre (MDRC), Wilfrid Laurier University, Waterloo, ON, Canada

**Keywords:** bimanual coordination, motor control disorders, dopamine, bradykinesia, hypokinesia, Parkinson’s disease

## Abstract

Bradykinesia is a well-documented DOPA-responsive clinical feature of Parkinson’s disease (PD). While amplitude deficits (hypokinesia) are a key component of this slowness, it is important to consider how dopamine influences both the amplitude (hypokinesia) and frequency components of bradykinesia when a bimanually coordinated movement is required. Based on the notion that the basal ganglia are associated with sensory deficits, the influence of dopaminergic replacement on sensory feedback conditions during bimanual coordination was also evaluated. Bimanual movements were examined in PD and healthy comparisons in an unconstrained three-dimensional coordination task. PD were tested “off” (overnight withdrawal of dopaminergic treatment) and “on” (peak dose of dopaminergic treatment), while the healthy group was evaluated for practice effects across two sessions. Required cycle frequency (increased within each trial from 0.75 to 2 Hz), type of visual feedback (*no vision*, *normal vision*, and *augmented vision)*, and coordination pattern (symmetrical in-phase and non-symmetrical anti-phase) were all manipulated. Overall, coordination (mean accuracy and standard deviation of relative phase) and amplitude deficits during bimanual coordination were confirmed in PD participants. In addition, significant correlations were identified between severity of motor symptoms as well as bradykinesia to greater coordination deficits (accuracy and stability) in PD “off” group. However, even though amplitude deficits (hypokinesia) improved with dopaminergic replacement, it did not improve bimanual coordination performance (accuracy or stability) in PD patients from “off” to “on.” Interestingly, while coordination performance in both groups suffered in the *augmented vision* condition, the amplitude of the more affected limb of PD was notably influenced. It can be concluded that DOPA-responsive hypokinesia contributes to, but is not directly responsible for bimanual coordination impairments in PD. It is likely that bimanual coordination deficits in PD are caused by the combination of dopaminergic system dysfunction as well as other neural impairments that may be DOPA-resistant or related to non-dopaminergic pathways.

## Introduction

Bradykinesia is a well-documented clinical feature of Parkinson’s disease (PD) that is characterized by progressive reduced frequency and/or amplitude (hypokinesia) of movements (1, 2). Bradykinesia responds well to dopamine replacement (2, 3), with amplitude (hypokinesia) being a key contributor to these improvements (4). Several researchers have demonstrated that, in addition to bimanual coordination deficits (i.e., decreased accuracy and/or stability), slower and smaller movements were also present during auditory-cued bimanually coordinated movements in PD (5–8). The presence of bimanual coordination deficits in individuals with PD supports the notion that the basal ganglia contribute to the execution of coordinated movements. Furthermore, based on these previous studies, it is plausible that bimanual coordination deficits in individuals with PD may be caused by amplitude and/or frequency deficits. If this were the case, one might predict that dopaminergic replacement therapy in individuals with PD should improve these components of bradykinesia and consequently coordination. Interestingly, since auditory cues have been demonstrated to influence only temporal aspects of auditory-cued movement (9), and given that previous coordination studies (mentioned above) have identified amplitude deficits in metronome-cued coordination tasks, we hypothesized that DOPA-responsive amplitude deficits in PD may have a significant contribution to cued bimanual coordination.

Although an association between the dopaminergic system, bradykinesia, and bimanual coordination deficits is possible, several studies have shown slower (10) and smaller movements (6, 10, 11) during bimanual coordination in individuals with PD independent of coordination impairments., yet this has not been verified with a true within-subject “on”–“off” dopaminergic study. In our previous study, it was demonstrated that slowness in the time needed to plan a switch between coordination phase patterns but not coordination in itself was improved with dopamine replacement (12). This study provided support that dopaminergic pathways may differentially influence slowness in planning from slowness in execution of bimanual coordination. While it is known that bradykinesia may refer to slowness in programing or executing a movement (13), the current study intended to clarify whether specific spatiotemporal aspects of bradykinesia during coordinated movement (such as amplitude) would also respond to dopaminergic treatment, without influencing coordination itself. It is possible that the deficits in coordination cannot fully be explained by dopaminergic system dysfunction since the basal ganglia are part of a distributed network involved in bimanual coordination that includes the supplementary motor area (SMA), cerebellum, primary motor cortex, premotor cortex, cingulate cortex, and primary sensorimotor area identified through functional imaging research (14–18). As a consequence, dopaminergic system dysfunction may only have a small contribution to bimanual coordination impairments in individuals with PD and alternatively, may be associated with disperse neural impairments.

Coordination impairments in PD may also be caused by sensorimotor integration deficits (19, 20). To support this viewpoint, Schettino et al. (21) manipulated visual feedback, object shape, and dopamine replacement while PD participants coordinated a unimanual reach-to-grasp movement. It was found that PD participants had slower movements and disrupted intralimb coordination during hand preshaping that was related to deficits in integrating proprioceptive and visual information compared to healthy older adults. Furthermore, dopamine replacement improved the speed of movement but did not ameliorate hand preshaping, suggesting that coordination impairments may be caused by a deficit in the integration of visual and proprioceptive information that are independent of the hypodopaminergic pathways (21). Thus, it is important to consider how sensory integration deficits may be influenced by the dopaminergic system during coordinated movements.

Alternatively, increased attentional and cognitive demands may negatively influence coordination performance as suggested by Almeida et al. (22). Increased attentional demands may involve performing anti-phase coordination (23, 24) or the combination of anti-phase with the presence of external auditory cueing (5). The negative relationship between attention and coordination could be related to difficulties in shifting attention or limited attentional resources that have been proposed for individuals with PD when performing simultaneous tasks (12, 25, 26). Importantly, it has been argued that executive dysfunction related to attention may be mediated by neural mechanisms that are not responsive to dopamine replacement (27–29). Research by Riekkinen et al. (30) compared the effects of dopamine replacement and noradrenalin (clonidine) replacement on different attention tasks in individuals with PD. It was found that dopamine replacement improved the speed of movement but had no effect on attention itself, leading the authors to conclude that attentional processes are not influenced by dopamine replacement in PD (30). Although bradykinesia improves with dopamine replacement, it is possible that bimanual coordination deficits may be caused by other neural impairments related to attention or sensorimotor integration pathways that cannot be corrected with dopamine replacement. If this were true, then bimanual coordination deficits in individuals with PD would not be caused by slowness or amplitude deficits. Thus, a secondary aim of the current study was to evaluate how conditions that might be associated with sensory-attentional networks might respond to dopamine replacement during coordination.

The primary objective of the current study was to determine the contribution of the dopaminergic system to bimanual coordination in individuals with PD while challenging various neural pathways through the manipulation of visual feedback, coordination pattern, and required cycle frequency. Manipulating these factors allowed the evaluation of two hypotheses: (1) If dopaminergic system dysfunction causes coordination deficits then coordination impairments would be observed in association with frequency or amplitude deficits in individuals with PD compared to healthy older participants after withdrawal of dopamine replacement (PD “off”). Importantly, dopamine replacement would improve frequency of movement and/or amplitude and coordination impairments in individuals with PD; (2) If bimanual coordination deficits were related to other neural impairments such as attention or sensorimotor integration pathways, then manipulating the sensory feedback to be attended to, to maintain the required cycle frequency and/or coordination demands would result in impairments in coordination performance in individuals with PD, independent of frequency or amplitude deficits and subsequently dopamine replacement.

## Materials and Methods

### Participants

Initially, 15 (*n* = 15, mean age = 68 ± 6.3, range 52–77 years) adults with a confirmed diagnosis of idiopathic PD were recruited to participate in this study. PD patients were diagnosed according to UK Brain Bank Criteria as confirmed by a movement disorders specialist, as well as each patient’s personal neurologist (at the time of enrollment into our research center’s database, see below). In addition, 11 age-matched (*n* = 11, mean age = 64.8 ± 6.4, range 55–75) participants without any neurological impairment were investigated as healthy comparisons. All individuals were right-hand dominant based on responses to the Waterloo Handedness Questionnaire (WHQ) (31). To verify that all individuals had the cognitive ability to perform the experiment and were free from dementia, they were assessed on the Modified Mini Mental State Examination (3-MS) (32) with previously verified cut-off score of 81 out of 100 (33) (see Table 1 for demographic information including 3-MS scores). All PD (mean 3-MS = 94.1 ± 5.2, range 83–100) and healthy comparison participants (mean 3-MS = 96.7 ± 3.8, range 89–100) scored above the criterion.

**Table 1:** **Demographic information of healthy comparison and PD participants**.

Participant	Group^1^	Age (in years)	Gender^2^	3-MS (out of 100)^3^	Education (in years)	Time between session (in min)^4^
1	PD	52	F	100	14	90
2	PD	62	F	96	12	90
3	PD	72	M	96	16	75
4*	PD	77	M	98	20	70
5	PD	67	M	91	12	75
6	PD	67	F	92	10	70
7	PD	76	M	93	12	70
8*	PD	69	M	88	10	90
9	PD	63	F	100	20	75
10*	PD	66	F	98	12	70
11*	PD	72	M	100	21	75
12	PD	65	F	98	15	70
13	PD	75	F	83	10	70
14	PD	67	F	91	15	70
15	PD	70	M	88	18	85
16	HC	74	M	100	18	65
17	HC	65	F	99	16	75
18	HC	63	F	100	15.5	70
19	HC	75	F	93	16	70
20	HC	67	M	97	14	80
21	HC	58	M	92	15	75
22	HC	62	M	99	18.5	80
23	HC	68	F	89	10	70
24	HC	58	F	100	16	70
25	HC	68	M	100	12	70
26	HC	55	F	95	16	70

*^1^PD, Parkinson’s disease participants; HC, healthy comparison participants*.

*^2^M, male; F, female*.

*^3^3-MS represents the modified Mini Mental State Examination*.

*^4^Time between sessions is equivalent to time “on” medication for PD participants*.

*Denotes PD participants that were included in correlation analyses but removed from all other analyses of coordination, movement amplitude, and frequency.

Parkinson’s disease participants were assessed for motor severity on the motor subsection of the Unified Parkinson’s Disease Rating Scale (UPDRS-III) (34) both with (“on”) and without (“off”) dopamine replacement. Since the goal of the current study was to evaluate the influence of bradykinesia (frequency and amplitude deficits) on bimanual coordination, four PD participants with the lowest overall severity as well as bradykinesia were excluded from coordination, amplitude, and frequency analyses (see Table 2, participants 4, 8, 10, and 11). These participants were removed to avoid mild and potentially low DOPA-responsive clinical features that would have confounded the primary objective of the current study. As a consequence, a sub-sample of 11 PD participants (*n* = 11, mean age = 66.9 ± 6.7, range 52–76 years) were included in coordination, amplitude, and frequency analyses. In addition to evaluating motor severity, the UPDRS-III was also used to confirm responsiveness of motor signs to dopaminergic medications in PD patients. All individuals with PD were confirmed to have a minimum five-point difference in motor severity as assessed between “off” and “on” scores on the UPDRS-III. Assessment “off” (mean UPDRS-III = 30.6 ± 8.6, range 18.5–46) occurred after overnight withdrawal from all dopaminergic treatments (mean time “off” = 14.9 ± 1.8 h, range 12–18). After completion of the first session, PD participants self-administered their regular dosage of medications and were re-examined on the UPDRS-III to represent “on” state (mean time “on” = 76.3 ± 8.1 min, range 70–90). Total daily levodopa equivalent dose (LED) were calculated for each participant as previously suggested (35). To determine which upper limb was more affected by PD, upper limb laterality scores were calculated and compared for both limbs from items 20–25 on the UPDRS-III that evaluates upper limb motor symptoms, similar to previous research (36–38). Based on these laterality scores, individuals with PD were also classified as bilaterally affected if both sides summed to five (or above) or were separated by less than one point. This was necessary to allow completion of any analyses comparing the amplitude and frequency deficits associated with the more and/or less affected limb. Session two was then completed in their “on” state (mean UPDRS-III = 20.0 ± 7.9, 10–35.5) (see Table 2 for clinical characteristics). Furthermore, to investigate if practice effects were present between the first and second sessions, all of the healthy comparison participants also performed two sessions (mean time between = 72.3 ± 4.7 min, range 65–80).

**Table 2:** **Clinical characteristics of PD participants**.

Participant	Duration since diagnosis (in years)^1^	Duration since first reported symptoms (in years)^1^	Dopamine medications^2^	Total daily levodopa equivalent dose (LED) (mg)^3^	Time “off” medication (h)	UPDRS-III “off” (out of 108)^4^	Time “on” medication (min)	UPDRS-III “on” (out of 108)^4^	Difference in UPDRS “off” and “on”	Disease laterality^3^
1	9	10	LD–CD	900	13.5	27	90	13.5	13.5	R < L
2	5	6	LD–CD	1000	12	46	90	35.5	10.5	L < R
3	8	10	LD–CD	300	15.5	30.5	75	22	8.5	L < R
4*	4	6	LD–CD	200	14.5	20	70	12.5	7.5	L < R
5	11	11	LD–CD, ras, pram	1450	13.5	31	75	22	9	R < L
6	6	6	LD–CD	900	18	42.5	70	22.5	20	R < L
7	9	9	LD–CD, tri, pram	1200	15	38	70	30.5	7.5	L < R
8*	1	3	LD–CD	300	15	21	90	14.5	6.5	L < R
9	2	6	LD–CD	400	12.5	32	75	23	9	L < R
10*	8	11	LD–CD, ent, ras	499	16.5	18.5	70	10	8.5	L < R
11*	0.5	1	LD–CD, ent	1023	16	21.5	75	12.5	9	L < R
12	4	5	LD–CD, pram	275	14.5	34	70	23	11	R < L
13	4	5	LD–CD	400	13.5	41.5	70	30.5	11	R < L
14	4	4	LD–CD	300	17	26.5	70	10.5	16	L < R
15	5	6	rop, ras	340	17	29.5	85	17.5	12	R < L

*^1^Information obtained from patient history on database. Duration since diagnosis was always reported. Duration since first symptoms was reported as duration since diagnosis if not reported differently by patient*.

*^2^LD–CD, levodopa–carbidopa (l-DOPA/DOPA decarboxylase inhibitor); ras, rasagiline (MAO-B selective agent); pram, pramipexole (dopamine receptor agonist); ent, entacapone (COMT inhibitors); rop, ropinirole (dopamine receptor agonist); tri, trihexyphenidyl (antimuscarinic)*.

*^3^Total daily levodopa dose (LED) was calculated using the formulas provided by Tomlinson et al. (35)*.

*^4^UPDRS-III scores represent clinical evaluation on the motor subsection of the Unified Parkinson’s Disease Rating Scale. Disease laterality was based on the sum of scores on the right side compared to the left side*.

*Denotes PD participants that were included in correlation analyses but removed from all other analyses of coordination, movement amplitude, and frequency.

Individuals were excluded from the study if they had any recent injury to their upper limbs that would influence their ability to perform the task, uncorrected vision (including uncorrected macular degeneration, cataracts, or glaucoma) or uncorrected hearing. Additionally, participants were excluded if they had previous history of stroke or serious brain trauma. Individuals with PD were included regardless of tremor, dyskinesia, or freezing but as previously mentioned, patients with low severity were excluded to avoid excessively mild motor signs (i.e., bradykinesia). All PD participants were recruited from the patient database at the Sun Life Financial Movement Disorders Research and Rehabilitation Centre (MDRC) at Wilfrid Laurier University. Healthy comparison participants were recruited from family and friends of the PD participants. Ethics for this study was granted from the Research Ethics Board (REB) at Wilfrid Laurier University. Written consent was obtained from all participants prior to initiation of experimental testing.

### Apparatus

**Figure 1:**
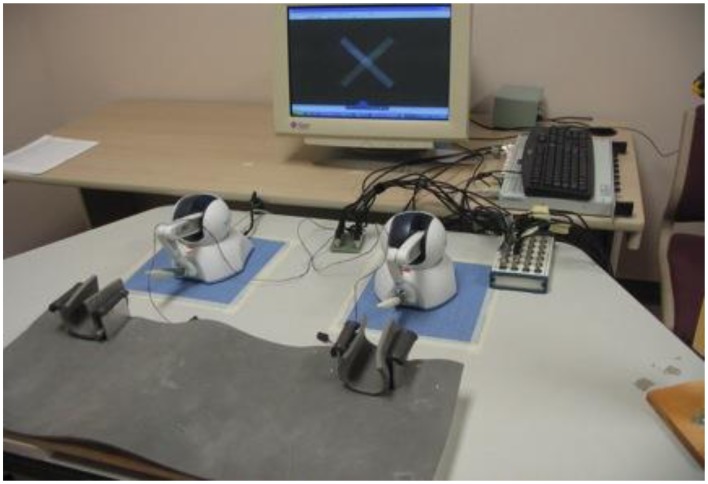
**Experimental set-up including Phantom Omni Devices, *forearm constraints*, and computer monitor with augmented feedback display**. During testing, individuals forearms were held in place by the forearm constraints lined with foam padding and grasped the white and blue pen-shaped attachment in their hand with their thumbs on top.

To perform the bimanual wrist flexion-extension movements, two robotic Phantom Omni haptic devices (SensAble Technologies Inc., Woburn, MA, USA) were used according to a previously published protocol [see Figure 1 and refer to (12) for full description of the apparatus].

### Protocol

Each participant performed the protocol in two sessions within a single day. Participants coordinated their wrists in flexion-extension movements as previously described [please see (12) for specific details of the typical protocol employed in our lab, including practice trials details].

Before each trial began, participants were instructed to coordinate their limbs in either in-phase or anti-phase. In-phase and anti-phase have both been shown to be intrinsic, stable coordination patterns commonly performed by the human motor system and have often been used to evaluate bimanual coordination from the perspective of motor control (39–43). In-phase was performed as a symmetrical pattern that required simultaneous flexion and extension of the wrists. This coordination required the synchronized use of homologous muscles in both limbs and a relative phase goal of 0° or 360° (44). Anti-phase was performed as an asymmetrical pattern that had participants simultaneously flex one wrist and extend the other wrist. This phase pattern required the use of non-homologous musculature in each limb and a relative phase goal of 180° (44).

Visual feedback was manipulated to permit three sensory feedback conditions: (i) *no vision* eliminated vision by blindfolding participants; (ii) *normal vision* allowed participants to see their wrist movements with the Omni devices; (iii) *augmented vision* eliminated vision of the moving limbs by covering the arms and required participants to use the augmented visual feedback on the computer monitor.

The combination of phase and sensory manipulations created six conditions. Each condition was performed in a pseudo-randomized order three times for a total of 18 trials per session. Each testing session lasted approximately 1.5 h including set-up, UPDRS-III and 3-MS assessments, and testing protocol. Rest was provided when needed to reduce fatigue.

### Data processing, dependent measures, and analysis

The customized software program (Matlab R2007b) recorded and stored displacement data from all three dimensions at a rate of 1000 Hz per second from each of the Omni devices. Data analysis was performed on medial-lateral displacement using a customized script (Matlab R2007b).

While there were instances where coordination during anti-phase became very uncoordinated and approached in-phase, there was rarely any clear and sustained spontaneous phase transitions (based on evaluation of the displacement data). This has been commonly reported, and thus, none of these trials would have been removed from analysis. However, freezing episodes did occur slightly more frequently. Approximately 36 trials involved clear freezing episodes across all participants, and hence were excluded from analysis. In order to evaluate and analyze the freezing episodes more closely, as well as the conditions that may have led to freezing episodes, further analysis (including EMG during episodes) will be required and reported elsewhere (since they do not contribute to the objectives of the current manuscript).

#### Coordination accuracy and stability

The calculation of the relative phase (position of one limb relative to the other) was used to evaluate coordination accuracy and stability. The relative phase was determined from the position of one limb relative to the other using the well-described formula (39):

$$\text{Relative phase}\;\left(\theta \right)=\tan-1\left[\left(dXR∕\mathrm{dt}\right)∕\mathrm{XR}\right]$$

However, since phase relationships could range from 0 to 360°, a linear transformation was performed on the relative phase to obtain values from 0 to 180° using the formula:

$$\text{New}\;\text{relative}\;\text{phase}\;\left(\theta n\right)=180-\left(\text{relative}\;\text{phase}\;\left(\theta \right)-180\right)$$

Absolute error (AE) of the relative phase (θ*n*) was calculated from the absolute difference between the average relative phase and the intended movement phase. An intended in-phase pattern would be quantified by a relative phase of 0° and an anti-phase pattern would be represented by 180° of relative phase. Mean and standard deviation of the AE of θ*n* was determined for each cycle frequency interval during every trial then calculated across groups.

#### Amplitude

The amplitude of each limb was measured independently to evaluate the spatial component of the movement. Specifically, this measure was used to evaluate if any amplitude deficits, representative of the hypokinesia component of bradykinesia, existed in individuals with PD. The amplitude was determined from each cycle of movement using the formula:

$$\begin{array}{llll} \text{Amplitude}\;\left(\text{cm}\right) & =\text{Amplitude}\;\text{Peakmax}\;\left(\text{cm}\right)\; & & \;\\ & \;-\text{Amplitude}\;\text{Peakmin}\left(\text{cm}\right)\; & & \; \end{array}$$

The mean amplitude of each limb was determined from averaging the amplitude of each peak during each cycle frequency interval for every trial then averaged across each group.

#### Performed cycle frequency

The performed cycle frequency of each limb was calculated to evaluate the temporal component of the movement. This measure was used to evaluate if any frequency deficits, representative of frequency component of bradykinesia, existed in individuals with PD. The performed cycle frequency was calculated using the movement cycles (positive to subsequent positive peak) during a given time interval using the formula:

$$\text{Cycle}\;\text{frequency}\left(\text{Hz}\right)\text{ = number}\;\text{of}\;\text{cycles/time}\;\left(\text{s}\right)$$

The mean performed cycle frequency of each the right and left limb was determined at each cycle frequency interval for every trial then averaged across each group.

#### Statistical analyses

Statistical analyses were performed using Statistica 8 (StatSoft Inc., Tulsa, OK, USA). *t*-Tests were performed on age, 3-MS scores, education, and time between sessions to verify that if any differences existed between PD and healthy comparison participants. Additionally, a paired *t*-test was performed on UPDRS-III scores of PD “off” and PD “on.”

In order to evaluate if a relationship existed between motor symptom severity and coordination performance, separate Pearson correlational analyses compared mean coordination accuracy and stability of relative phase for their association with motor symptom severity in both PD “off” and “on” (see Table 4). In addition, Pearson correlational analyses were also performed to evaluate the association between bradykinesia (score derived from items 23, 24, 25, 26, and 31 of the UPDRS-III) and mean coordination performance (accuracy and stability of relative phase) for both PD “off” and “on.” Mean coordination accuracy and stability were calculated across all manipulations of conditions, phase, and performed cycle frequency for each PD participant. In contrast to analyses of coordination, amplitude, and frequency, all 15 PD participants were included in the correlations since the goal of these analyses was to evaluate how disease severity was associated to coordination performance.

To analyze coordination accuracy (mean relative phase) and stability (standard deviation of relative phase), a mixed-model (between and within-group) ANOVA was designed with group (PD, healthy comparisons) as the between-group factor and within-group factors: session (one, two), condition (*no vision, normal vision, augmented vision*) × phase (in-phase, anti-phase) × required cycle frequency (0.75, 1, 1.25, 1.5, 1.75, 2 Hz). Mean amplitude and performed cycle frequency were analyzed using a mixed-model ANOVA designed with between-group factors (PD, healthy comparisons) and within-group factors: session (one, two) × limb (more affected/matched limb, less affected/matched limb) × condition (*no vision, normal vision, augmented vision*) × phase (in-phase, anti-phase) × required cycle frequency (0.75, 1, 1.25, 1.5, 1.75, 2 Hz). Limb was treated as a factor allowing comparison between the more and less affected limbs in PD (see Table 2) and matched hands in healthy comparison group. Hands were matched based on age and gender when possible.

In order to evaluate our specific hypotheses, planned comparisons were made for the dependent variables (see Table 3). Specifically, to determine the effects of basal ganglia dysfunction on coordination performance, amplitude, and frequency, planned comparisons were performed on session 1 using group (PD “off,” healthy comparisons) as the between-group factor. To evaluate the effects of dopamine replacement on coordination accuracy, coordination stability, amplitude, and frequency, planned comparisons were performed between session one (PD “off”) and session two (PD “on”) of PD participants. Finally, a planned comparison was performed on session one compared to session two of healthy comparison participants based on the overall mixed-model design to determine if any practice effects existed for coordination accuracy and stability. A complete list of all between- and within-group factors for each dependent measure in each planned comparison is presented in Table 3.

**Table 3:** **Description of statistical comparisons**.

ANOVA planned comparisons	Dependent measures	Between-group factor	Within-group factors	Dependent measures	Between-group factor	Within-group factors
PD “off” vs. healthy comparisons	Coordination accuracy and coordination stability	Group	Condition, phase, required cycle frequency	Amplitude and performed cycle frequency	Group	Limb, condition, phase, required cycle frequency
PD “off” vs. PD “on”	Coordination accuracy and coordination stability	N/A	Session, condition, phase, required cycle frequency	Amplitude and performed cycle frequency	N/A	Session, condition, phase, required cycle frequency
HC session 1 vs. session 2	Coordination accuracy and coordination stability	N/A	Session, condition, phase, required cycle frequency			

In all cases, an alpha level of 0.05 was used to define statistical significance. Tukey’s HSD *post hoc* analysis was used to investigate any significant interactions that were revealed from the ANOVA analyses.

## Results

### Comparison of demographic variables

Student’s *t*-tests revealed that there were no significant differences between PD and healthy comparison participants when considering age, self-reported education, amount of time between sessions and 3-MS scores (see Table 4). The UPDRS-III scores were found to be significantly different (mean difference = 11.6 ± 3.7) between PD “off” (mean UPDRS “off” = 34.4 ± 6.6) and PD “on” (mean UPDRS “on” = 22.8 ± 7.4) [*t*(10) = 10.5, *p* < 0.001].

**Table 4:** **Statistical comparisons of age, education, 3-MS, and time between sessions of PD and healthy comparison (HC) participants**.

	PD (*n* = 11)	HC (*n* = 11)	*t* Statistic (df) and *p*-value
Age (in years)	66.9 (±6.7)	64.8 (±6.43)	*t*(20) = −0.74, *p* = 0.46
3-MS (out of 100)	93.5 (±5.2)	96.7 (±3.9)	*t*(20) = 1.67, *p* = 0.11
Self-reported education (in years)	14.0 (±3.2)	15.2 (±2.5)	*t*(20) = 0.97, *p* = 0.34
Time between sessions (in min)	76.4 (±8.1)	72.3 (±4.7)	*t*(20) = −1.5, *p* = 0.16

### Pearson correlational analyses between motor symptom severity and bradykinesia to coordination performance (accuracy and stability)

**Figure 2:**
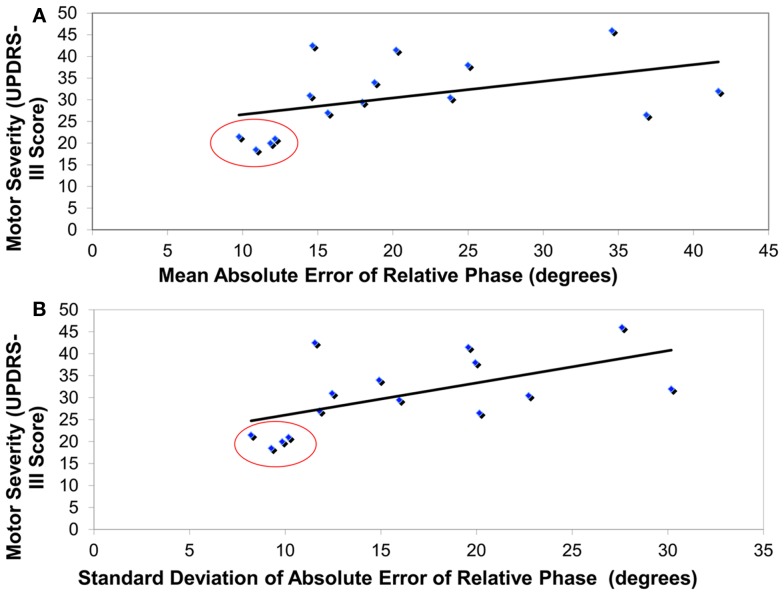
**Correlationanalyses revealing that higher motor severity in PD patients “off” (as measured by UPDRS-III) was associated with (A) greater coordination error (absolute error of relative phase, degrees) and (B) greater coordination variability (standard deviation of absolute error of relative phase, degrees)**. Red circles highlight the four least severe PD patients that were removed from all other analyses.

A significant correlation was revealed between the severity of motor symptoms (as revealed by UPDRS-III) in PD “off” participants and the mean coordination accuracy across all conditions [*r*(15) = 0.48, *p* < 0.05] (see Figure 2A). In addition, the mean variability in coordination across all conditions was significantly correlated to the degree of severity of motor symptoms in PD “off” [*r*(15) = 0.58, *p* < 0.01] (see Figure 2B). Furthermore, significant correlations were identified between overall bradykinesia scores and both mean coordination accuracy [*r*(15) = 0.78, *p* < 0.01] and mean variability in coordination [*r*(15) = 0.83, *p* < 0.01] across all conditions. In contrast, the correlations between motor symptom severity or bradykinesia and coordination performance in PD “on” were not significant.

### PD “off” vs. healthy comparison participants

All significant main (and interaction) effects that are superseded by significant interaction effects are not described in detail. In addition, only significant interactions involving group are presented herein. Please see Table A1 (coordination accuracy and stability) and Table A3 (amplitude and performed cycle frequency) in Appendix for complete statistical results of significant main effects and interactions. Interactions that did not reach significance are not reported.

#### Coordination accuracy

**Figure 3:**
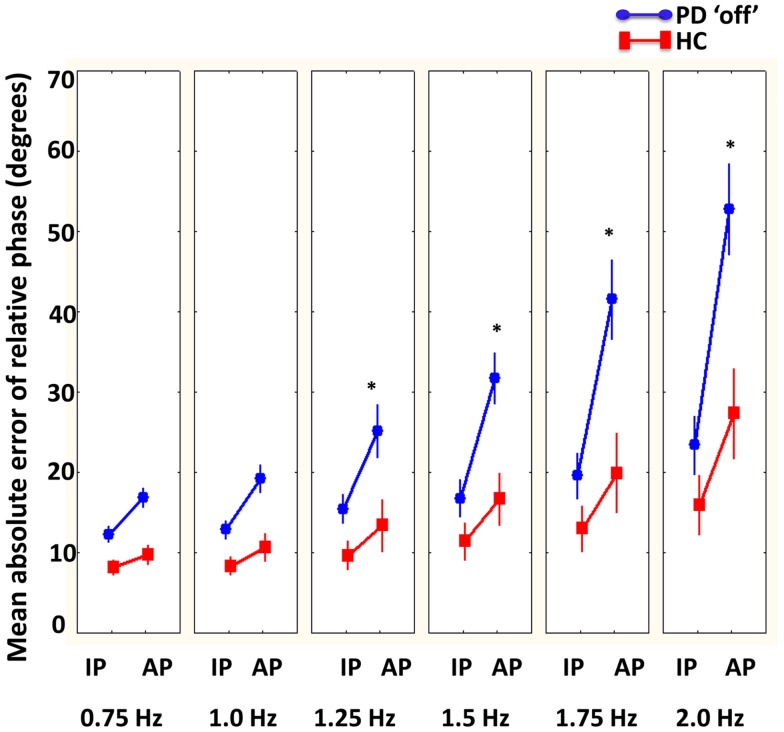
**Mean absolute error of relative phase (degrees) compared between PD “off” and healthy comparison (HC) participants as a function of phase (in-phase = IP and anti-phase = AP) and cycle frequencies**. Results showed that HC had more accurate coordination compared to PD “off” participants at faster cycle frequencies (1.25–2 Hz) in anti-phase (bars denote standard error). *Denotes significant differences between HC and PD “off” participants.

A significant interaction between group, phase, and required cycle frequency was revealed [*F*(5, 100) = 2.83, *p* < 0.05]. As illustrated in Figure 3, Tukey’s *post hoc* analysis revealed that healthy comparison participants had greater coordination accuracy in anti-phase at the fastest required cycle frequencies (1.75 and 2 Hz) compared to PD “off” participants. Additionally, PD “off” participants had greater coordination accuracy during in-phase at 0.75 and 1 Hz compared to 2 Hz and during anti-phase at 0.75 and 1 Hz compared to 1.5, 1.75, and 2 Hz. In contrast, healthy comparison participants had greater coordination accuracy during anti-phase at 0.75 and 1 Hz compared to 2 Hz but no difference in coordination accuracy during in-phase coordination at any required cycle frequency. In addition, a significant main effect of condition was found [*F*(2, 40) = 5.41, *p* < 0.01]. Tukey’s *post hoc* analysis demonstrated greater coordination accuracy when coordinating in *no vision* and *normal vision* relative to the *augmented vision* condition.

#### Coordination stability

A significant interaction between group and phase was revealed [*F*(1, 20) = 10.19, *p* < 0.01]. Tukey’s *post hoc* revealed that both PD “off” and healthy comparison participants had more variable coordination in anti-phase compared to in-phase, but the healthy participants had significantly less variable coordination in anti-phase compared to PD “off.” In addition, there were also a significant interaction for group, condition, and required cycle frequency [*F*(10, 200) = 2.45, *p* < 0.01]. Tukey’s *post hoc* did not reveal any significant differences between PD “off” and healthy comparison participant but revealed that there was less variability at the two slowest required cycle frequencies (0.75 and 1 Hz) and coordination became increasingly more variable at each subsequent required cycle frequency interval particularly in *augmented vision* relative to *no vision* and *normal vision*.

#### Mean amplitude (affected/less affected limbs PD “off” compared to matched limbs)

**Figure 4:**
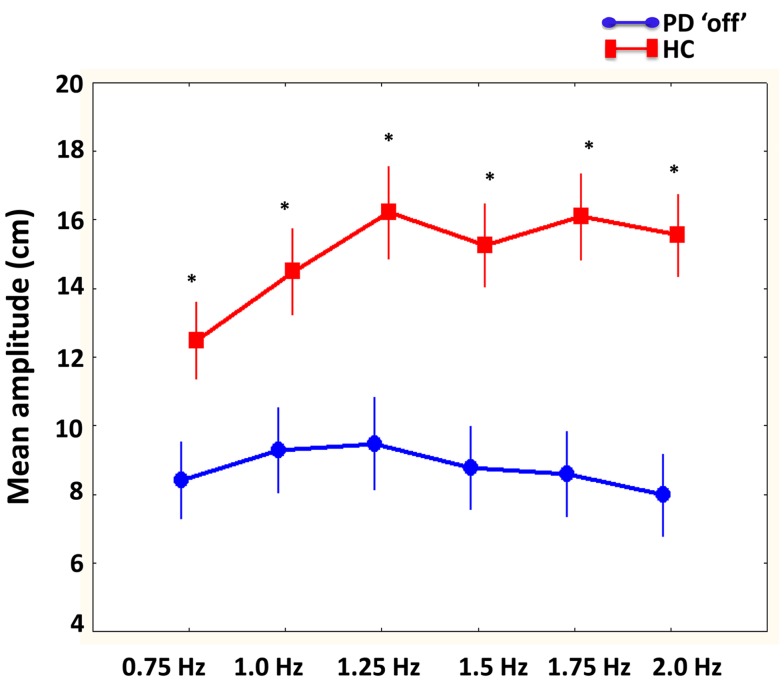
**Mean amplitude (cm) of limb movements compared between PD “off” and healthy comparison (HC) participants as a function of cycle frequencies**. Results showed that larger movement were produced by HC participants compared to PD “off” at all cycle frequencies.

There were a significant interaction between group and required cycle frequency [*F*(5, 100) = 8.0, *p* < 0.001]. As illustrated in Figure 4, Tukey’s *post hoc* analysis showed that the healthy older participants were performing larger movements with both limbs compared to PD “off,” particularly at the fastest required cycle frequencies (1.25, 1.5, 1.75, and 2 Hz). Interestingly, PD “off” did not change amplitude regardless of increasing required cycle frequency. There was also a significant main effect of phase [*F*(1, 20) = 14.8, *p* < 0.01] demonstrating larger amplitude movements were produced with both limbs during in-phase compared to anti-phase coordination.

Disease laterality in PD participants was shown to affect amplitude as revealed by a significant interaction between group, limb, and condition [*F*(2, 40) = 17.9, *p* < 0.01]. Tukey’s *post hoc* analysis showed that the less affected limb in PD participants had larger movements in all conditions compared to the more affected limb. In addition, both groups had larger movements in both limbs in *normal vision* compared to *no vision*, and *no vision* compared to *augmented vision*.

#### Mean performed cycle frequency (affected/less affected in PD “off” compared to matched hands in healthy comparisons)

There was a significant interaction between group, limb, and required cycle frequency [*F*(5, 100) = 3.5, *p* < 0.01]. Tukey’s *post hoc* analysis indicated that the performed cycle frequency in both limbs was not different at any required cycle frequency between PD “off” and healthy comparisons. In both groups, as required frequency increased, the cycle frequency in both limbs increased except healthy control participants maintained cycle frequency between 1.25 and 1.5 Hz in both limbs.

### PD “off” vs. PD “on”

All significant main (and interaction) effects that are superseded by significant interaction effects are not described in detail. In addition, only significant interactions involving session are presented herein. Please see Table A2 (coordination accuracy and stability) and Table A4 (amplitude and performed cycle frequency) in Appendix for complete statistical results of significant main effects and interactions. Interactions that did not reach significance are not reported.

#### Coordination accuracy

A main effect of condition was found [*F*(2, 20) = 4.2, *p* < 0.05] that revealed coordination was more accurate in *normal vision* compared to *augmented vision*. There was no significant influence (main effect or interactions) of dopamine replacement on coordination accuracy.

#### Coordination stability

A significant main effect of condition was found [*F*(2, 20) = 3.7, *p* < 0.05] that demonstrated coordination was less variable with *normal vision* compared to *augmented vision*. Similar to coordination accuracy, there was no influence of dopamine replacement (significant main effect or interactions) on coordination variability.

#### Mean amplitude (More affected compared to less affected)

**Figure 5:**
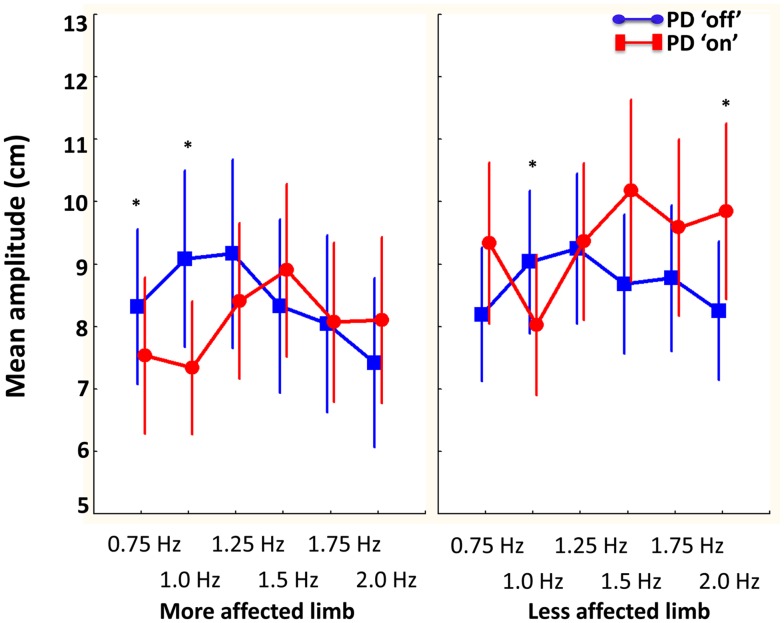
**The influence of dopamine replacement on mean amplitude (cm) of the more and less affected limb in PD participants across cycle frequencies**. Results demonstrated that PD “on” had larger amplitude movements at the faster cycle frequency in the less affected limb. However, PD “off” had larger amplitude movements in the more and less affected limb at slower cycle frequencies (0.75–1 and 1 Hz, respectively) (bars denote standard error). *Denotes significant differences between PD “off” and PD “on.”

The influence of disease laterality and dopamine replacement on amplitude was revealed by a significant interaction between session, limb, and required cycle frequency [*F*(5, 50) = 2.8, *p* < 0.05]. As illustrated in Figure 5, Tukey’s *post hoc* analysis indicated that PD “off” produced larger movements in the most affected limb at slower cycle frequencies (0.75 to 1.25 Hz) compared to PD “on.” Similarly, larger movements were produced in the least affected limb at 1 Hz in PD “off” compared to PD “on.” In contrast, at the fastest cycle frequency (2 Hz) PD “on” had larger amplitude movements in the less affected limb compared to PD “off.” PD “off” produced larger movements in the least affected limb at all required cycle frequencies compared to the more affected limb. In contrast, PD “on” produced larger movements in the least affected limb at the faster cycle frequencies (1.25–2 Hz) compared to the more affected limb.

#### Mean performed cycle frequency (More affected compared to less affected)

Unlike amplitude, dopamine replacement did not have an influence (significant main effect or interactions) on performed cycle frequency of either limb.

### Healthy comparison participants – effects of practice

All significant main (and interaction) effects that are superseded by significant interaction effects are not described in detail. In addition, only significant interactions involving session are presented herein. Please see Table A5 in Appendix for all significant main effects and interactions. Interactions that did not reach significance are not reported.

#### Coordination accuracy

No significant influence (main effect or interactions) of sessions was revealed for coordination accuracy in the healthy older participants.

#### Coordination stability

There was a significant interaction between session and condition [*F*(1, 10) = 4.3, *p* < 0.05]. Tukey’s *post hoc* analysis revealed that there was less variability when coordinating with *augmented vision* in session two compared to session one. Additionally, coordination was less variable during *normal vision* compared to *augmented* vision in session one but not session two.

## Discussion

The primary objective of the current study was to evaluate the DOPA-responsiveness of specific spatiotemporal aspects of movement (such as amplitude and frequency) that contribute to movement slowness during bimanual coordination. Previous research demonstrated that the ability to initiate a switch between coordinated movements was improved with dopamine replacement (12). Given that PD typically presents with unilateral amplitude and frequency of movement deficits (which are generally responsive to dopamine replacement), it was hypothesized that if bimanual coordination deficits in PD were primarily related to amplitude (hypokinesia) and/or frequency deficits, that both coordination and these clinical features would be ameliorated with dopaminergic replacement. Alternatively, if deficits in bimanual coordination were associated with other DOPA-resistant or non-dopaminergic neural impairments, then amplitude and frequency components of movement would be expected to respond to dopamine alone, while deficits in coordination would be independent DOPA-responsive clinical features of movement.

The main finding of the current study was that both bimanual coordination impairments and movement amplitudes deficits were identified between PD “off” compared to healthy comparison participants. However, even though these amplitude deficits (hypokinesia) improved with dopaminergic treatment during clinical assessment, and also while coordinating the less affected limb at faster cycle frequencies, bimanual coordination impairments did not. Taken together with our previous findings, dopamine appears to improve both limb amplitude during coordinated movement (i.e., hypokinesia), as well as the speed at which a switch in plans for different coordination patterns can be achieved, while initiation and the bimanual coordination in itself does not improve. It should be noted however that correlational analyses in the current study revealed that bimanual coordination deficits were associated with the severity of dopaminergic system dysfunction as well as overall bradykinesia, suggesting that disease progression and bradykinesia contribute, but are not primarily responsible for bimanual coordination impairments in PD. The results support our alternative hypothesis that DOPA-resistant neural impairments (or potentially impairments in non-dopaminergic pathways) have a key contribution to bimanual coordination deficits in PD.

### Dopaminergic system and bradykinesia (hypokinesia) during coordinated movements in PD

Typically, anti-phase compared to in-phase coordination is performed with greater error and variability at the faster cycle frequencies in adults (41, 45). The current study found that PD “off” dopaminergic treatment had significantly worse coordination accuracy during anti-phase at faster cycle frequencies (1.75 and 2 Hz) as well as more variable coordination in anti-phase compared to healthy older adults. These results support previous findings that individuals with PD have greater difficulty coordinating their limbs in anti-phase particularly as frequency of movement approaches 2 Hz as compared to young and older adults (5, 7, 11, 24). Imaging research examining anti-phase coordination in PD demonstrated different connectivity as well as hypoactivation in the SMA-proper as a consequence of dopamine loss (46). As suggested by Johnson et al. (24), the difficulty for individuals with PD to bimanually coordinate in asymmetrical anti-phase movements may be associated to the disruption in the thalamocortical pathway to the SMA leading to a breakdown in the precise sequential muscle activation of homologous muscles. Thus, understanding how the amplitude and frequency components of the movement were affected may help to clarify the breakdown in coordination in PD.

Since no differences were identified between individuals with PD and healthy for the movement frequency of each limb, we can conclude that PD maintained the appropriate cycle frequency (i.e., performed cycle frequency) similar to healthy. This is in agreement with previous studies that have found that auditory cueing (compared to removal of cues) is useful for maintaining the correct cycle frequency of movement in PD during bimanual coordination (11, 24). In addition, auditory cues were found to result in less variable cycle times, amplitudes, and coordination during bimanual circle drawing tasks in PD, young, and elderly participants (47). It has been argued that individuals with PD have an impaired ability to internally regulate timing of repetitive movements especially at faster frequencies (36, 48–51). In view of the fact that individuals with PD were maintaining the correct cycle frequency with the metronome, it would suggest that attention was directed at synchronizing movements with the external cues to compensate for internal timing deficits. Based on the current results, external timing cues were sufficient to reduce the effects of bradykinesia (i.e., slowness) and as a consequence, dopamine replacement did not influence the performed cycle frequency during bimanually coordinated movements in individuals with PD. Thus, the frequency component of bradykinesia does not appear to contribute to coordination deficits in PD during externally cued bimanual coordination.

In regards to amplitude of movement, PD “off” had significantly smaller amplitudes compared to healthy participants predominantly at faster cycle frequencies (1.25–2 Hz). Furthermore, significant correlations were identified between overall bradykinesia and coordination impairments in PD “off.” This would suggest that amplitude deficits may have contributed to coordination impairments in PD. However, a key novel finding of the current study was that even though amplitude in the less affected limb and amplitude modulation was improved with dopamine replacement (representative of improvements in bradykinesia with dopamine replacement during movement execution), neither coordination accuracy nor stability was influenced by dopaminergic modulation. In the current study, healthy elderly participants increased the amplitude of their movements with increased cycle frequency but PD “off” patients did not follow this trend. PD “off” participants maintained similar amplitudes of movement as cycle frequency increased. In healthy adults, simultaneously increases in amplitude of movements are often observed with cycle frequency increases due to the greater forces and velocities being applied by the faster moving limbs (52, 53). Considering the performed cycle frequency was modulated appropriately, the amplitude modulation of movements appears to be constrained in the PD “off” group during auditory-cued bimanual movements. However, even in the PD “on” group where amplitude modulation was improved a mismatch between limbs still existed. The deficits in amplitude modulation as well as different movement amplitudes in the limbs likely contributed to an amplitude interference effect that has been previously reported in bimanual coordination tasks in young healthy adults when they required to produce different movement amplitudes in each limb (54, 55). Thus, even when dopaminergic treatment in PD “on” improves the size of movements in the least affected limb at faster cycle frequencies and improves amplitude modulation, the amplitude mismatch between the limbs is still present in PD likely contributing to coordination impairments in PD.

The lack of contribution of dopamine replacement to coordination performance is in agreement with the hypothesis that bimanual coordination impairments are influenced by a distributed neural network (14–18) rather than a direct and causal relationship to limb asymmetries in the amplitude of bimanually coordinated movements. It was previously suggested that specific aspects of movements speed (such as amplitude) may normalize with dopaminergic treatment, while parameters that depend on precise and different patterns of neuronal firing such as timing and coordination are not restored with dopamine replacement (56). Thus, other DOPA-resistant neural impairments related to PD likely contribute to bimanual coordination deficits in PD patients. Since PD is known to affect many different neural pathways outside the dopaminergic system such as the cholinergic, noradrenergic, and serotonergic systems throughout its’ progression as revealed by the Braak system (57), it is likely that these neural impairments also contribute to bimanual coordination deficits in PD.

### Relationship between attention, sensorimotor integration, and coordination in PD

It was proposed as an alternative hypothesis, that bimanual coordination deficits in individuals with PD may be associated with a more disperse neural dysfunction involving attentional or sensorimotor integration processes. Since PD participants were able to maintain the required cycle frequency for each limb in the current task, it is likely that attentional resources were directed to the external auditory cues. Furthermore, anti-phase coordination has been shown to require a greater attentional load than in-phase coordination (58). It is possible that the presence of these external auditory cues particularly during anti-phase coordination negatively influenced coordination performance in PD as suggested by our previous research (5, 12). It has previously been suggested that individuals with PD may have difficulty shifting attention or limited attentional resources during simultaneous tasks (12, 25, 26). Research has shown that attentional impairments are resistant to dopaminergic treatment but respond to clonidine (noradrenaline) in PD (30). It may be possible that individuals with PD lack the attentional resources or ability to shift attention at the faster cycle frequencies with external cueing when having to perform anti-phase coordination.

Furthermore, the manipulation of sensory feedback conditions allowed the investigation of whether sensorimotor integration deficits might contribute to coordination deficits in PD. The results of the current study did not provide conclusive evidence to support sensorimotor integration problems as a key influence on bimanual coordination. Although coordination performance (both accuracy and variability) was worse, with smaller movement amplitudes during the *augmented vision* condition (compared to *normal vision* and *no vision* conditions), no coordination differences existed between groups. Furthermore, the more affected limb in PD patients produced smaller amplitude movements compared to the least affected limb, particularly in *augmented vision*, but a similar effect was not reflected in coordination performance. Thus, while specific aspects of movement control appear to be affected by the availability of sensory feedback, coordination does not appear to be directly affected by sensory feedback manipulations.

Difficulties in sensorimotor integration in PD are usually observed during slow voluntary movements (20) such as self-paced reach-to-grasp movements (21, 59) or fast internally regulated rhythmic movements (56), rather than fast externally cued movements used in the current study. It was expected that individuals with PD would demonstrate greater deficits compared to healthy older adults using augmented visual feedback since previous research has demonstrated that variability of coordination in PD participants did not improve after training with augmented visual feedback (60). Furthermore, PD have been shown to undershoot the amplitude of movement when they are unable to see their moving limb (61) as was the case with the augmented visual feedback used in the current study. Furthermore, PD patients have marked deficits in visuo-motor coordination that has been linked to disrupted parietal-premotor circuits (62, 63). However, augmented visual feedback led to similar impairments in coordination in PD and healthy older adults suggesting that it was not the cause of coordination deficits in PD. Since both PD and healthy older adults had near equal difficulty in coordinating with augmented visual feedback, it is likely that all individuals have difficulty making visuo-motor transformations of the limbs into a single representation of their coordinated movements.

It is important to note that healthy older adults demonstrated a significant improvement in coordination variability in the *augmented vision* condition across sessions. Unlike healthy older adults, PD participants were unable to benefit from augmented visual feedback. No improvements were seen in PD across sessions regardless of practice or dopamine replacement. It has previously been suggested that PD are impaired in learning (28, 64–66). Furthermore, it was argued that learning is influenced by dopaminergic modulation (64, 66). Feigin et al. (66) found that learning motor sequences was impaired in PD patients after administration of dopamine replacement. Jahanshahi et al. (64) proposed that the tonic release of dopamine with dopamine replacement therapy impairs the phasic release of dopamine that is essential for learning. Impaired motor learning in individuals with PD could be an alternative explanation for the current results, in the sense that motor learning was not able to occur between sessions due to the administration and subsequent influence of dopamine replacement. However, PD participants did not demonstrate more variable coordination in the *augmented vision* condition relative to healthy elderly participants, which suggests that this did not contribute to the current results. Nevertheless, the influence of dopamine replacement on motor learning needs to be carefully considered in future research when examining PD patients across sessions.

### Potential neural mechanisms of bimanual coordination impairments in PD

The neural mechanism that may be responsible for bimanual coordination impairments during cued anti-phase coordination are likely complex due to the distributed neural networks that are involved in these types of movements (15, 16). Traditionally, impairments in anti-phase bimanual coordination have been associated with hypoactivation in the SMA due to basal ganglia dysfunction (24, 46). However due to the distributed neural network involved in bimanual coordination, dysfunction in pathways linking the basal ganglia to other neural structures such as the anterior cingulate cortex (ACC) or dorsolateral prefrontal cortex (DLPFC) may also be involved. The ACC has been argued to be involved in inhibiting unwanted or competing movement responses during bimanual movements (15). In this view, dysfunction in the pathways involving the ACC may lead to a decreased ability to inhibit competing responses during bimanual coordination such as homologous muscles in anti-phase coordination. Another possible candidate is the attentional system. Imaging research has identified that PD have decreased functional connectivity between DLPFC and basal ganglia with the SMA during anti-phase coordination (46). The DLPFC has been associated with the frontoparietal (top–down) attentional system, that includes the superior frontal cortex and intraparietal cortex, that is involved in the preparation and selection of stimuli and responses that have a previous goal (67). In addition, the DLPFC has been shown to be involved in inhibiting irrelevant sensory inputs (68). Deficits in attention have been shown in individuals with PD that has been associated to degeneration of noradrenergic cells as improvement in neuropsychological tests of attention were observed with clonidine treatment in PD (30). Furthermore, decreased cholinergic function related to degeneration of the nucleus basilis of Meynert (nbM) and pedunculopontine (PPN) nuclei that supply the cerebral cortex and thalamo-striatal has been associated with attentional dysfunction in PD (69). Importantly, synucleinopathic degeneration of these cells precedes the nigral dopaminergic cell loss that is the hallmark of PD pathology (57). The association between these deficits and bimanual coordination is difficult to untangle since several systems are likely involved in contributing to coordination deficits in PD. Under normal circumstances of bimanual coordination, individuals internally regulate attentional resources to the sensory inputs (auditory cues, proprioceptive, and visual information) to make appropriate responses. In addition, the sensory information received allows for the appropriate responses to be selected and unwanted responses to be inhibited. However, dysfunction in the regulation of attention to the appropriate sensory information as well as inhibition of unwanted sensory information leads to inappropriate responses. This may in fact may have lead to the manifestation of amplitude impairments in our PD patients that contributing to deficits in bimanual coordination. This would be particularly evident during fast externally paced anti-phase coordination when several sources of sensory information are available as the sensory information needs to be processed more quickly with a greater attentional load. Thus, the impairment is several neural pathways involving the basal ganglia, ACC, DLPFC, and SMA likely all contribute to deficits in cued bimanual anti-phase coordination when the frequency of movement is increased and multiple sources of sensory feedback are required to produce temporally and spatially precise movements.

There are several limitations of the current study. Firstly, measures of attention were not directly examined in the current sample of PD patients. As a consequence, the association between impairments in attention and deficits in bimanual coordination in PD is theoretical in nature. Future studies should directly examine attention as well as the amount of cholinergic and noradrenergic dysfunction to verify if these associations to bimanual coordination impairments in PD patients are warranted. Secondly, the cued dynamic increase in frequency within trials was sufficient to circumvent the frequency impairments (bradykinesia) in our PD patients. Although the current study was more focused on determining the relationship between DOPA-responsive amplitude (hypokinesia) and coordination impairments, future studies that are concerned with DOPA-responsive frequency impairments (bradykinesia) may benefit from evaluating self-paced rather than externally cued movements. Finally, individuals with low motor severity were removed from the majority of our analyses limiting the generalizability of our findings to more severe PD patients. However, based on our correlational analyses that found significant correlations between the degree of coordination impairments and motor severity as well as bradykinesia, caution should be used in future bimanual coordination research that includes individuals with PD with mild motor symptom severity.

### Conclusion

The current results support that the dopaminergic system’s influence over specific spatiotemporal aspects of movement (such as unilateral amplitude deficits) do not directly influence bimanual coordination performance. Based on the current results, the demand on attentional resources imposed by anti-phase coordination and external auditory cueing may be important factors to consider for their contribution to bimanual coordination deficits in PD. Under certain conditions, internal timing deficits during self-paced movements, impairments in sensorimotor integration (when limiting compensatory sensory feedback), and/or dysfunctional attentional processes may all contribute to bimanual coordination deficits in PD. Collectively, this would suggest that disperse neural impairments are likely responsible for the coordination impairments in PD that involve to some extent dopaminergic system dysfunction, but also DOPA-resistant neural impairments outside the dopaminergic system.

## Conflict of Interest Statement

The authors declare that the research was conducted in the absence of any commercial or financial relationships that could be construed as a potential conflict of interest.

## Appendix

**Table A1:** **Statistical results from ANOVA analysis on planned comparison between PD “off” and healthy comparison (HC) participants and between PD “off” and PD “on” to evaluate the effects of basal ganglia dysfunction on coordination accuracy and stability**.

Factor(s)	Coordination accuracy	Main significant finding	Coordination stability	Main significant finding
Group	*F*(1, 20) = 12.46, *p* < 0.01*	Greater accuracy by HC	*F*(1, 20) = 12.29, *p* < 0.01*	Less variability by HC
Condition	*F*(2, 40) = 5.4, *p* < 0.01*	Greater accuracy in no vision and normal vision	*F*(2, 40) = 9.46, *p* < 0.001*	Less variability in no vision and normal vision
Phase	*F*(1, 20) = 24.36, *p* < 0.001*	Greater accuracy in in-phase	*F*(1, 20) = 58.98, *p* < 0.001*	Less variability in in-phase
Required cycle frequency	*F*(5, 100) = 45.47, *p* < 0.001*	Greater accuracy at slower frequencies (0.75, 1, and 1.25 Hz)	*F*(5, 100) = 44.08, *p* < 0.001*	Less variability at slower frequencies (0.75, 1, and 1.25 Hz)
Group × phase	*F*(1, 20) = 5.39, *p* < 0.05*	Greater accuracy by HC during anti-phase	*F*(1, 20) = 8.51, *p* < 0.01*	Less variability by HC during anti-phase
Group × required cycle frequency	*F*(5, 100) = 4.34, *p* < 0.01*	Greater accuracy by HC at fastest cycle frequencies (1.75 and 2 Hz)	*F*(5, 100) = 1.85, *p* = 0.11	N/A
Group × phase × required cycle frequency	*F*(5, 100) = 2.83, *p* < 0.05*	See Section “Coordination Accuracy” under Section “PD ‘Off’ vs. Healthy Comparison Participants”	*F*(5, 100) = 2.03, *p* = 0.08	N/A
Group × condition × required cycle frequency	*F*(10, 200) = 0.4, *p* = 0.9	N/A	*F*(10, 200) = 2.54, *p* < 0.01*	See Section “Coordination Stability” under Section “PD ‘Off’ vs. Healthy Comparison Participants”

* denotes significance below p < 0.05

**Table A2:** **Statistical results from ANOVA analysis on planned comparison between PD “off” and PD “on” to evaluate the effects of dopaminergic treatment on coordination accuracy and stability**.

Factor(s)	Coordination accuracy	Main significant finding	Coordination stability	Main significant finding
Session	*F*(1, 10) = 0.75, *p* = 0.4	N/A	*F*(1, 10) = 0.0.3, *p* = 0.9	N/A
Condition	*F*(2, 20) = 4.2, *p* < 0.05*	See Section “Coordination Accuracy” under Section “PD ‘Off’ vs. PD ‘On”’	*F*(2, 20) = 3.66, *p* < 0.05*	See Section “Coordination Stability” under Section “PD ‘Off’ vs. PD ‘On”’
Phase	*F*(1, 10) = 23.93, *p* < 0.001*	Greater accuracy in in-phase	*F*(1, 10) = 56.97, *p* < 0.001*	Less variability in in-phase
Required cycle frequency	*F*(5, 50) = 25.46, *p* < 0.001*	Greater accuracy at slower frequencies (0.75, 1, and 1.25 Hz)	*F*(5, 50) = 26.39, *p* < 0.001*	Less variability at slower frequencies (0.75, 1, and 1.25 Hz)

*** denotes significance below p < 0.05

**Table A3:** **Statistical results from ANOVA analysis on planned comparison between PD “off” and healthy comparison (HC) participants to evaluate the effects of basal ganglia dysfunction on amplitude and frequency of movements**.

Factor(s)	Amplitude	Main significant finding	Cycle frequency	Main significant finding
Group	*F*(1, 20) = 14.0, *p* < 0.01*	Larger amplitudes by HC	*F*(1, 20) = 0.01, *p* = 0.9	N/A
Limb	*F*(1, 20) = 0.07, *p* = 0.8	N/A	*F*(1, 20) = 0.2, *p* = 0.7	N/A
Condition	*F*(2, 40) = 15.17, *p* < 0.001*	Larger amplitudes in normal vision vs. no vision and no vision vs. augmented vision	*F*(2, 40) = 1.56, *p* = 0.2	N/A
Phase	*F*(1, 20) = 14.8, *p* < 0.01*	Larger amplitudes in in-phase	*F*(1, 20) = 5.19, *p* < 0.05*	Faster frequencies in in-phase
Required cycle frequency	*F*(5, 100) = 11.04, *p* < 0.001*	Smaller amplitude movements at slowest frequency (0.75 Hz)	*F*(5, 100) = 232.17, *p* < 0.001*	Cycle frequencies increased as required frequency increased (except maintenance between 1.25 and 1.5 Hz)
Group × required cycle frequency	*F*(2, 40) = 8.00, *p* < 0.001*	See Section “Mean Amplitude (Affected/Less Affected Limbs PD ‘Off’ Compared to Matched Limbs)”	*F*(2, 40) = 0.66, *p* = 0.7	N/A
Group × limb × condition	*F*(2, 40) = 5.69, *p* < 0.01*	See Section “Mean Amplitude (Affected/Less Affected Limbs PD ‘Off’ Compared to Matched Limbs)”	*F*(2, 40) = 0.034, *p* = 1.0	N/A
Group × limb × required cycle frequency	*F*(5, 100) = 2.13, *p* = 0.07	N/A	*F*(5,100) = 3.51, *p* < 0.01*	See Section “Mean Performed Cycle Frequency (Affected/Less Affected in PD ‘Off’ Compared to Matched Hands in Healthy Comparisons)”

* denotes significance below p < 0.05

**Table A4:** **Statistical results from ANOVA analysis on planned comparison between PD “off” and PD “on” to evaluate the effects of dopaminergic treatment on amplitude and frequency of movements**.

Factor(s)	Amplitude	Main significant finding	Cycle frequency	Main significant finding
Session	*F*(1, 10) = 0.46, *p* = 0.5	N/A	*F*(1, 10) = 0.49, *p* = 0.5	N/A
Limb	*F*(1, 10) = 4.68, *p* = 0.06	N/A	*F*(1, 10) = 0.35, *p* = 0.6	N/A
Condition	*F*(2, 20) = 3.76, *p* < 0.05*	Larger amplitudes in normal vision vs. augmented	*F*(2, 20) = 1.13, *p* = 0.3	N/A
Phase	*F*(1, 10) = 1.19, *p* = 0.3	N/A	*F*(1, 10) = 2.39, *p* = 0.2	N/A
Required cycle frequency	*F*(5, 50) = 3.87, *p* < 0.01*	Larger amplitudes at 1.25 vs. 1 Hz	*F*(5, 50) = 102.54, *p* < 0.001*	Cycle frequencies increased as required frequency increased (except maintenance between 1.25 and 1.5 Hz)
Session × required cycle frequency	*F*(5, 50) = 5.1, *p* < 0.001*	Larger amplitudes by PD “off” at slowest frequencies (0.75 and 1 Hz)	*F*(5, 50) = 0.59, *p* = 0.7	N/A
Session × limb × condition	*F*(2, 20) = 0.55, *p* = 0.6	N/A	*F*(2, 20) = 0.28, *p* = 0.8	N/A
Session × limb × required cycle frequency	*F*(5, 50) = 2.78, *p* < 0.05*	See Section “Mean Amplitude (More Affected Compared to Less Affected)”	*F*(5, 50) = 0.52, *p* = 0.8	N/A

* denotes significance below p < 0.05

**Table A5:** **Statistical results from ANOVA analysis on planned comparison between session 1 and session 2 of healthy comparison (HC) participants to evaluate the effects of practice on coordination accuracy and stability**.

Factor(s)	Coordination accuracy	Main significant finding	Coordination stability	Main significant finding
Session	*F*(1, 10) = 0.01, *p* = 0.9	N/A	*F*(1, 10) = 18.31, *p* < 0.01*	Less variability in session 2
Condition	*F*(2, 20) = 9.18, *p* < 0.01*	Greater accuracy in no vision and normal vision	*F*(2, 20) = 11.4, *p* < 0.001*	Less variability in no vision and normal vision
Phase	*F*(1, 10) = 14.6, *p* < 0.01*	Greater accuracy in in-phase	*F*(1, 10) = 20.51, *p* < 0.01*	Less variability in in-phase
Required cycle frequency	*F*(5, 50) = 33.64, *p* < 0.001*	Less accuracy at fastest frequency (2 Hz)	*F*(5, 50) = 51.03, *p* < 0.001*	More variability at fastest frequency (2 Hz)
Session × condition	*F*(2, 20) = 0.38, *p* = 0.7	N/A	*F*(2, 20) = 4.28, *p* < 0.05*	See Section “Coordination Stability” under Section “Healthy Comparison Participants – Effects of Practice”

* denotes significance below p < 0.05
